# Influence of Mechanical Circulatory Support on Endothelin Receptor Expression in Human Left Ventricular Myocardium from Patients with Dilated Cardiomyopathy (DCM)

**DOI:** 10.1371/journal.pone.0169896

**Published:** 2017-01-17

**Authors:** Florian Gärtner, Getu Abraham, Astrid Kassner, Daniela Baurichter, Hendrik Milting

**Affiliations:** 1 Erich & Hanna Klessmann Institute for Cardiovascular Research and Development, Clinic for Thoracic and Cardiovascular Surgery, Heart and Diabetescenter NRW, Bad Oeynhausen, Germany; 2 Institute of Pharmacology, Pharmacy and Toxicology, University of Leipzig, Leipzig, Germany; Scuola Superiore Sant'Anna, ITALY

## Abstract

**Background:**

In terminal failing hearts ventricular assist devices (VAD) are implanted as a bridge to transplantation. Endothelin receptor (ETR) antagonists are used for treatment of secondary pulmonary hypertension in VAD patients. However, the cardiac ETR regulation in human heart failure and during VAD support is incompletely understood.

**Methods:**

In paired left ventricular samples of 12 dilated cardiomyopathy patients we investigated the density of endothelin A (ET_A_) and B (ET_B_) receptors before VAD implantation and after device removal. Left ventricular samples of 12 non-failing donor hearts served as control. Receptor quantification was performed by binding of [^125^I]-ET-1 in the presence of nonselective and ET_A_ selective ETR ligands as competitors. Additionally, the ETR mRNA expression was analyzed using quantitative real-time-PCR.

**Results:**

The mRNA of ET_A_ but not ET_B_ receptors was significantly elevated in heart failure, whereas total ETR density analyzed by radioligand binding was significantly reduced due to ET_B_ receptor down regulation. ET_A_ and ET_B_ receptor density showed poor correlation to mRNA data (spearman correlation factor: 0.43 and 0.31, respectively). VAD support had no significant impact on the density of both receptors and on mRNA expression of ET_A_ whereas ET_B_ mRNA increased during VAD. A meta-analysis reveals that the ET_A_ receptor regulation in human heart failure appears to depend on non-failing hearts.

**Conclusions:**

In deteriorating hearts of patients suffering from dilated cardiomyopathy the ET_A_ receptor density is not changed whereas the ET_B_ receptor is down regulated. The mRNA and the proteins of ET_A_ and ET_B_ show a weak correlation. Non-failing hearts might influence the interpretation of ET_A_ receptor regulation. Mechanical unloading of the failing hearts has no impact on the myocardial ETR density.

## Introduction

Endothelins have been described as potent vasoconstrictor peptides consisting of 21 amino acids secreted by the endothelium [1]. Three isoforms, designated ET-1, ET-2 and ET-3 have been identified [2]. ET-1, the principal isoform is secreted by many cell types beyond endothelial cells including cardiac myocytes [3, 4].

Endothelins mediate their activity mainly in an autocrine/paracrine manner [5, 6] on its receptors of which two have been identified, named endothelin receptor A (ET_A_) and B (ET_B_). Endothelin receptors are G-protein-coupled receptors and mediate their action essentially in a phospholipase C dependent manner. They are expressed in a variety of tissues including the myocardium.

The endothelin system is involved in cardiovascular functions. ET_A_ receptor expressed by vascular smooth muscle cells mediates prolonged vasoconstriction whereas the ET_B_ receptor which is located on endothelial cells is primarily considered to cause NO-mediated vasodilatation [7].

In the heart, endothelin contributes to contractility [8], chronotropy [9] and fibrosis [10] and induces myocyte hypertrophy [11]. Hypertension, pulmonary hypertension and heart failure is associated with changes of the endothelin system [12, 13]. Bosentan, a dual endothelin receptor antagonist is used for treatment of secondary pulmonary hypertension in patients with mechanical circulatory support (MCS) [14] or awaiting heart transplantation [15].

Ventricular assist devices (VAD) are implanted in terminal failing heart patients as a bridge for heart transplantation or as destination therapy. Cardiac regeneration under mechanical unloading by VAD was repeatedly reported [16, 17]. However, the cellular and molecular changes responsible for these salutary effects as well as the implication of the endothelin system in the cardiac amelioration are incompletely understood. Morawietz *et al*. found a normalization of increased ET_A_ receptor expression levels in 7 of 10 patients with end-stage heart failure after VAD unloading on mRNA level while ET_B_ expression remained unchanged [18]. However, data on the protein expression during VAD support are not available so far.

In this study, we investigated the expression of both endothelin receptors on the level of mRNA and protein in parallel in paired myocardial samples of dilated cardiomyopathy (DCM) patients receiving a VAD.

## Material and Methods

### Study cohort

Transmural left ventricular (LV) myocardium of 12 male patients with end stage non-ischemic DCM receiving a continuous flow VAD (DuraHeart, Heartmate II, HeartWare or VentrAssist, respectively) at the age of 46.8 ± 11.1 years was collected from the para-apical region during VAD implantation (VAD-IP) and later from the same patient at heart transplantation (VAD-HTx). Samples without macroscopic signs of fibrosis were snap frozen in liquid nitrogen and stored at -80°C until analysis. The time of VAD-support was 722 ± 436 days (mean ± SD). All patients provided written informed consent for the use of their cardiac tissue samples. For clinical data of the patients, see Table 1. LV myocardial samples were also obtained from non-failing (NF) donor hearts which could not be transplanted for diverse reasons. The donor cohort was sex (12 male) and age (47.0 ± 11.0 years) matched with DCM patients at the time of VAD-implantation. The ethics committee of the Ruhr University Bochum at the Heart and Diabetescenter NRW in Bad Oeynhausen, Germany, approved the study which conforms to the declaration of Helsinki (Vote No. 21/2013).

**Table 1:** Clinical data at the time of MCS-implantation and medication during VAD support.

patient No.	gender	disease	age [y]	LVEDD	LVESD	EF	CI	PCW	CVP	PAP	Medication duringVAD support
#1	M	DCM	41	n.a.	n.a.	20	2.7	20	18	n.a.	βB; MA; AI
#2	M	DCM	51	72	65	20	1.4	33	23	38	βB; MA; AI; EB
#3	M	DCM	59	61	56	20	1.3	27	10	32	n.a.
#4	M	DCM	30	n.a.	n.a.	15	n.a.	n.a.	n.a.	14	βB; MA; AI
#5	M	DCM	54	78	72	25	2.2	12	19	45	βB; MA; AI
#6	M	DCM	64	n.a.	n.a.	20	1.5	29	18	n.a.	βB; MA; AI
#7	M	DCM	32	59	57	10	1.8	15	n.a.	n.a.	MA; AI
#8	M	DCM	45	104	94	20	2.0	38	19	48	βB; MA; AI
#9	M	DCM	37	88	82	20	2.9	17	16	46	βB; MA; AI; EB
#10	M	DCM	55	75	67	27	2.0	n.a.	n.a.	n.a.	βB; MA; AI
#11	M	DCM	58	70	62	30	1.8	19	6	28	MA; EB
#12	M	DCM	35	80	72	20	2.1	23	8	33	βB; MA; AI
means (SD)			47 (11)	76 (13)	70 (11)	21 (5)	2.0 (0.5)	23 (8)	15 (5)	36 (11)	

**Abbreviations**: **AI** = ACE-inhibitor (enalapril or ramipril); **βB** = β-adrenergic receptor blocker (carvedilol or bisoprolol); **CI** = cardiac index [l min^-1^ m^-2^]; **CVP**° = °central venous pressure [mm Hg]; **DCM** = dilative cardiomyopathy; **EB** = endothelin receptor blocker (Bosentan); **EF** = ejection fraction (echo) [%]; **LVEDD** = left ventricular end diastolic diameter [mm Hg]; **LVESD** = left ventricular end systolic diameter [mm Hg]; **MA** = mineralocorticoid-receptor antagonist (spironolactone or eplerenone); **PAP** = pulmonary artery pressure [mm Hg]; **PCW** = pulmonary capillary wedge pressure [mm Hg].

### mRNA quantification

RNA was isolated from left ventricles using commercial kits (RNeasy, Qiagen, Hilden, Germany). Reverse transcription of myocardial RNA was done using 250 ng total RNA and 50 units Superscript II (Life Technologies GmbH, Darmstadt, Germany) after random priming with hexamers. mRNA of ET_A_ and ET_B_ receptors was quantified in duplicates with the ΔΔC_T_ method in a StepOne Plus (Applied Biosystems, Foster City, US-CA) using TaqMan probes labeled with FAM and TAMRA. Normalization was performed using the geometric mean of the three reference genes *RPL13A*, *TOP2B* and *TPT1*, which were selected from 10 candidate genes and validated using the Visual Basic Application geNorm (M values < 0.5) [19].

Sequences for primers and probes used for real-time experiments were: ET_A_ sense 5'-TGGTGTGCACTGCGATCTTC-3'; ET_A_ probe 5'-ACCCTCATGACTTGTGAGATGTTGA-3'; ET_A_ antisense 5'-GCAATTCTCAAGCTGCCATTC-3'; ET_B_ sense 5'-CCGCCACGCACCATCT-3'; ET_B_ probe 5'-CCTCCCCCGTGCCAAGGACC-3'; ET_B_ antisense 5'-TTGATGTATTTGAAAGTCTCCTTGATCT-3'; RPL13A sense 5'-TCACGAGGTTGGCTGGAAGT-3'; RPL13A probe 5'-CAGGCAGTGACAGCCACCCTGGA-3'; RPL13A antisense 5'-CTTGGCTTTCTCTTTCCTCTTCTC-3'; TPT1 sense 5'-AGCGCCGGCTATGCCCCTG-3'; TPT1 probe 5'-AGCGCCGGCTATGCCCCTG-3'; TPT1 antisense 5'-GTGATTACTGTGCTTTCGGTACCTT-3'; TOP2B sense 5'-GCTAAAAAGGGAAAACCGTCTTC-3'; TOP2B probe 5'-ATACAGTCCCTAAGCCCAAGAGAGCCCC-3'; TOP2B antisense 5'-GAGTTTACAGCCTCTACTACTTTCTTCTGTT-3'. PCR conditions were: 95°C, 10’; 95°C, 15”; 60°C, 1’; 40 cycles; 300 nM primer; 150 nM probe.

### Membrane preparation

150 mg of LV myocardium was minced with scissors and homogenized in 1 mL ice-cold homogenization buffer (50 mM Tris-HCl buffer, pH 7.4, with 1 mM EDTA and 2% BSA) containing a protease inhibitor cocktail (Sigma-Aldrich, St. Louis, US-MO, #P2714) using a Ribolyser (Peqlab Erlangen, Germany) for 20 seconds 3 times in 3-minute intervals. Samples were diluted to 20 mL with homogenization buffer and centrifuged at 700 g for 15 minutes. The supernatant was passed through four layers of cheesecloth and centrifuged at 50,000 g for 25 minutes. The pellet was washed once by resuspension in homogenization buffer without BSA and centrifugation and finally resuspended in resuspension buffer (39.8 mM HEPES, pH 7.4, 135 mM NaCl, 5.4 mM KCl, 0.5 mM MgCl_2_, 0.3 mM Na_2_HPO_4_, 1.2 mM CaCl_2_, 0.4 mM MgSO_4_, 4 mM NaHCO_3_) containing protease inhibitor cocktail. Protein content was determined using bicinchoninic acid and BSA as a standard. Membranes were diluted in incubation buffer (0.2% BSA in resuspension buffer) containing protease inhibitor to yield a protein concentration of 15 μg/40 μL and stored at -80°C.

### Radioligand binding studies

For determination of the total amount of endothelin receptors, competitive binding experiments were performed in a total of 1 mL incubation buffer. A constant amount of ≈10,000 cpm of [^125^I]-ET-1 in 100 μL was mixed with 6 different concentrations of unlabeled ET-1 (500 μL) as a competitor ranging from 2 to 486 pM in the final solution. Membranes were diluted 1:10 and 400 μL of the membrane suspension (15 μg of protein) was added to the ligands and incubated for 60 minutes at 37°C in siliconized (Sigmacote, Sigma-Aldrich) glass tubes in a shaking water bath. Binding was stopped by the addition of 14 mL incubation buffer. Bound ligand was separated by vacuum filtration through glass fiber filters (Millipore, Billerica, US-MA) presoaked with incubation buffer containing 4% BSA and a washing step with another 14 mL of incubation buffer. The activity of the filters was determined in a gamma counter (Multi Crystal LB2111, Berthold Technologies, Bad Wildbad, Germany). The total binding without competitor which yields the top plateau of the competition curve was determined without ET-1 in quadruplicates. Residual activity not displaced by 1 μM Bosentan was defined as nonspecific binding (bottom plateau of the competition curve) and determined in duplicates. For every sample the displacement experiment was repeated once. Analyses were performed using the software GraphPad Prism 6 (GraphPad Software, La Jolla, US-CA) and a nonlinear regression (One site–Fit log IC_50_). The receptor density (B_max_) was calculated as (B_0_ x IC_50_) / [L] [20]. B_0_ was obtained by subtracting the nonspecific binding from the total binding; IC_50_ was regarded as constant and set to 49.3 pM which was the mean IC_50_ value of all competition curves.

To calculate the ratio of ET_A_ and ET_B_, displacement was performed with 8 different concentrations of the ET_A_ selective antagonist BQ-123 ranging from 50 μM to 640 pM. Total binding and nonspecific binding was determined in duplicates each. The displacement experiment with BQ-123 was repeated once for every sample. The ratio of ET_A_ and ET_B_ receptors were calculated from a nonlinear regression (Two sites–Fit log IC_50_) using the software GraphPad Prism 6.

### Determination of collagen content using 4-hydroxyproline measurements

The collagen content in tissue samples was determined by aminoacid analysis of 4-hydroxyproline using the chloramin-T method [21]. Two independent tissue samples were weighted and hydrolyzed in 6 M HCl. Analysis was done in duplicate for each sample as published before [22].

### Determination of plasma Big-ET1

Plasma Big-ET1 before and 28 ± 11 (mean ± SD) days after VAD-implantation was measured using a commercially available ELISA according to manufacturer’s conditions (Biomedica, Vienna, Austria).

## Results

We quantified endothelin receptors by quantitative real-time-PCR in paired (pre- vs. post-VAD) myocardial samples of 12 VAD patients with DCM. In parallel the receptor density was quantified by radio ligand binding with [^125^I]-ET-1. The total receptor number was obtained according to DeBlasi *et al*. by competition with unlabeled ET-1 [20] (see S1 Fig for representative competition curves). For the assessment of the relative amount of the two endothelin receptor subtypes ET_A_ and ET_B_ the binding of the radioligand was inhibited by BQ-123. This resulted in biphasic competition curves (see S2 Fig for representative curves). We compared our findings to 12 rejected donor hearts which were matched according to gender and age.

**Fig 1:**
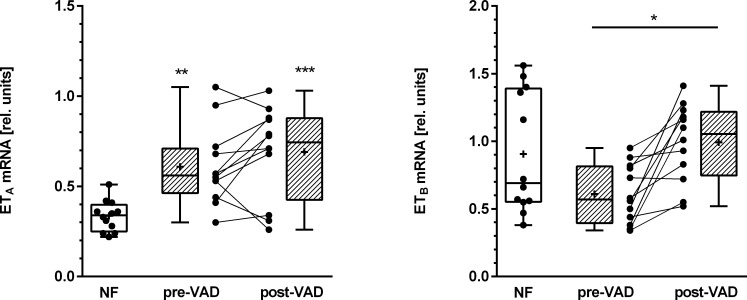
Relative quantification of endothelin receptor mRNA with quantitative real-time-PCR. mRNA from cardiac tissue was reversely transcribed and analyzed using TaqMan probes and primers specific for the ET_A_ receptor **(A)** or the ET_B_ receptor **(B)**, respectively. The geometric mean of the expression of RPL13A, TPT1 and TOP2B served as endogenous reference. Results were obtained in duplicates and analyzed using the ΔΔCT-method. Shown are the mean values. ** p < 0.01 and ***p < 0.001 compared to NF-samples using One Way ANOVA and Bonferroni’s multiple comparisons test.

The LV mRNA of the ET_A_ receptor was significantly higher (p < 0.001, one way ANOVA and Bonferroni’s multiple comparisons test) in the failing myocardium of patients compared to NF hearts (0.61 ± 0.22 vs. 0.34 ± 0.09 ([arbitrary units] means ± SD); Fig 1A). After VAD-support the elevated mRNA of ET_A_ (0.69 ± 0.25) was not changed in comparison to the time of device implantation and remained on a significantly (p < 0.01) elevated level compared to control myocardium. In contrast, we did not find different concentrations of ET_B_ mRNA in failing myocardium compared to rejected donor hearts (NF, pre-VAD: 0.91 ± 0.45, 0.61 ± 0.22; Fig 1B). However, the mRNA of ET_B_ was significantly upregulated after VAD (0.99 ± 0.29; p < 0.05). Thus, the ET_A_ receptor mRNA was increased in terminal failing hearts and not regulated by mechanical unloading, whereas the ET_B_ receptor mRNA was only regulated during VAD support but not different between NF and samples from failing hearts.

**Fig 2:**
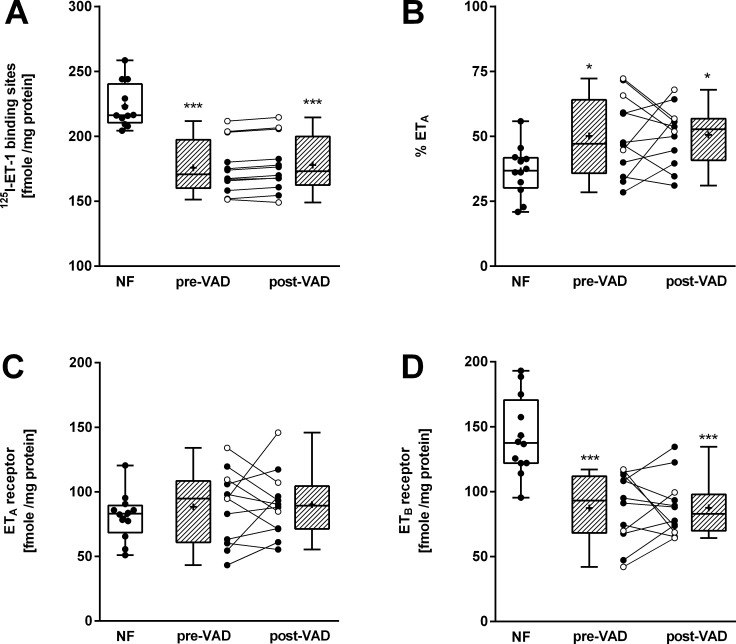
Expression of endothelin receptors determined by radioligand binding studies. **A)** Total amount of endothelin receptors in 12 paired myocardial samples from VAD-receivers (pre-VAD and post-VAD) and 12 non-failing (NF) donor hearts. Binding of [^125^I]-ET-1 was replaced by varying amounts of unlabeled ET-1. **B)** Receptor subtype analysis. For the assessment of the relative amount of receptor subtypes, [^125^I]-ET-1 was replaced by varying amounts of the selective ET_A_ receptor antagonist BQ-123. **C-D)** Density of the ET_A_ receptor **(C)** and the ET_B_ receptor **(D)** as calculated from both replacement experiments. All experiments were performed in duplicates. Shown are the mean values. During VAD-support, patients were treated (open circles) or not (closed circles) with Bosentan. * p < 0.05 and ***p < 0.001 compared to NF-samples using One Way ANOVA and Bonferroni’s multiple comparisons test.

Radioligand binding was done for total endothelin receptor binding (Fig 2A) and for receptor subtype analysis (Fig 2B). The mean K_D_ of [^125^I]-ET-1 was 49.3-pM ± 15.7 (SD). The K_D_ of NF, pre-VAD and post-VAD samples were not significantly different. Furthermore, there was no significant difference in the K_D_-values of patients receiving Bosentan or not. The ET_A_ receptor in pre-VAD samples (88.4 ± 28.0 fmole/mg protein) was not different from donor hearts (81.0 ± 18.4 fmole/mg protein; Fig 2C). We also did not observe an effect of mechanical unloading on the ET_A_ receptor density (post-VAD: 90.2 ± 25.2 fmole/mg protein). In contrast, we observed a significant lower expression of ET_B_ receptors in terminally failing hearts (p < 0.001 vs. NF; NF 142.6 ± 30.4 and pre-VAD: 87.4 ± 26.6 fmole/mg protein; Fig 2D). ET_B_ receptor expression was also not influenced by mechanical unloading (post-VAD: 87.6 ± 22.3 fmole/mg protein). Three patients received Bosentan during VAD support (see Table 1) which was far too low to draw any conclusion on an influence of this adjuvant therapy on the ET_A_ or ET_B_ receptor expression during mechanical unloading, respectively.

**Fig 3:**
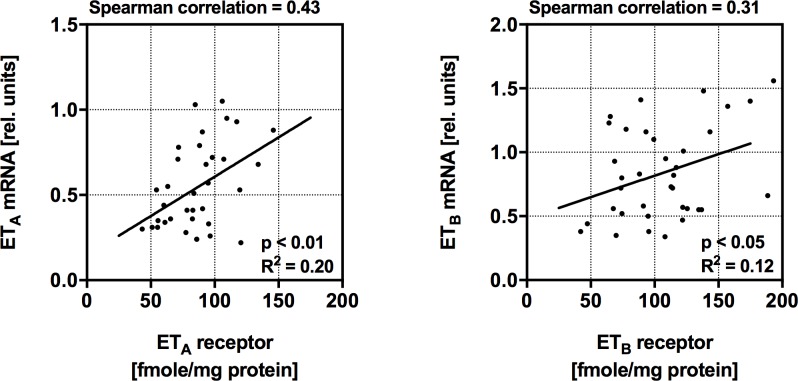
Correlation of mRNA and protein concentration. Samples of the same hearts were analyzed in parallel for mRNA and protein concentrationα of ET_A_ **(A)** and ET_B_ **(B)**. The spearman correlation coefficient and the results of the linear regression analysis are given.

The data from mRNA and protein analysis for both endothelin receptors measured in the same specimen were plotted for correlation tests. We found only a weak correlation with a R^2^ in the linear regression model of 0.20 and 0.12 for ET_A_ or ET_B_ receptors. We also compared the data by the non-parametric Spearman correlation tests which revealed coefficients of 0.43 and 0.31 for the ET_A_ or ET_B_ receptors, respectively (Fig 3).

We compared ET_A_ and ET_B_ receptor expression with the myocardial collagen content but did not find any evidence for a correlation (see S3 Fig).

Plasma Big-ET1 is significantly lower (p < 0.05, t-Test) after 28 ± 11 days of VAD-support compared to the time of VAD-implantation (see S4 Fig). There is no evidence for a correlation of myocardial ET_A_ or ET_B_ receptor density and plasma Big-ET1 at VAD-implantation or after VAD-support, respectively (see S5 Fig).

## Discussion

In our study, we investigated the ET_A_ and ET_B_ receptor mRNA expression as well as the density of endothelin receptors in LV samples of DCM patients. Furthermore, for the first time the effect of VAD unloading on cardiac endothelin receptor expression on mRNA as well as on protein level was analyzed in parallel.

As obtained by real-time-PCR, the mRNA of the ET_A_ receptor was up regulated in left ventricular pre-VAD samples, whereas the ET_B_ mRNA expression was unaltered compared to NF samples (Fig 1). In numerous previous studies the myocardial regulation of endothelin receptor mRNA in human heart failure was analyzed. The results, however, were not always concordant. ET_A_ receptor mRNA in the LV of DCM patients has been reported to be increased [18, 23] or not regulated [24–26], respectively. In the same manner, ET_B_ receptor mRNA was down regulated [25] or not altered [18, 23, 26] in LV DCM samples.

On the protein level, we found no alteration in the density of the ET_A_ receptor in LV DCM samples (pre-VAD) compared to the NF samples. However, the ET_B_ receptor density was significantly decreased in the ventricles of DCM patients explaining the reduced total endothelin receptor binding sites in this study (Fig 2). As it is the case for receptor mRNA, examination of literature concerning the density of ET_A_ and ET_B_ receptors in LV samples of DCM patients, yield inconsistent data [23, 25–30]. Some authors found no alteration in the density of ET_A_ receptors in heart failure [26, 27, 29] whereas others reported increased levels of myocardial ET_A_ binding sites [23, 25, 28, 30]. It appears that the study outcome is highly dependent on the control hearts which were used in the given study (see S6 Fig). NF hearts represent not well characterized tissue samples by their nature. In previous reports the control myocardium contained between 14 and 204 fmole/mg ET_A_ receptor. Of note, in studies where the control samples contained low densities of this receptor (between 14 and 42 fmole/mg) [23, 25, 28, 30] the DCM samples showed significantly increased numbers of endothelin receptors. Whereas in studies where the NF control hearts had values above 70 fmole/mg there was no significant change of ET_A_ receptor density in DCM hearts [26, 27, 29]. In our study, we found 81 fmole/mg ET_A_ receptors in NF control hearts and could not find significant changes of the ET_A_ receptor densities in patients with need for VAD support. Thus, our data on ET_A_ expression agree with previously reported receptor regulation in the human heart and do not suggest ET_A_ receptor regulation in DCM.

In contrast data from the ET_B_ receptor in the literature appear to be not dependent on the control hearts used although in the literature a remarkable scatter of data on the ET_B_ receptor density in NF myocardium was reported. We and others [23, 25, 29] found a significant down regulation of the ET_B_ receptor in the myocardium of patients with DCM whereas others [26–28, 30] did not find a significant regulation of the ET_B_ receptor in human heart failure. This discrepancy might be due to the comparably small number of control hearts used in some studies (n = 4 [30], n = 5 [27, 28], n = 6 [29] or n = 7 [26], respectively).

We did not find an effect of mechanical circulatory support on the regulation of endothelin receptor mRNA or protein. This is in accordance to mRNA data obtained by a global gene expression analysis of 30 paired samples from VAD-supported DCM hearts using Affymetrix HG-U133 Plus 2.0 arrays [31]. However, in a previous study Morawietz *et al*. found down regulation of increased mRNA during mechanical circulatory support [18]. Unfortunately, the authors investigated a mixed heart failure cohort containing DCM and ischemic cardiomyopathy (ICM) samples. Serneri *et al*. reported different regulation of endothelin receptors in patients with DCM or ICM, respectively [26]. In addition, patients in that study were supported by first generation pulsatile VADs, which might unload the ventricle stronger compared to continuous flow systems.

Endothelin receptor mRNA and protein showed only a weak correlation which might reflect that transcription and translation of these receptors are regulated on different levels (Fig 3). Endothelin receptor mRNA and protein and might have different half-lifes. An independent regulation of mRNA and protein was observed previously for the myocardial beta 1 adrenergic receptor [32] and other proteins [33]. It appears that the analysis of mRNA does not contribute significantly to the understanding of endothelin receptor regulation on the protein level.

It was discussed that endothelin receptor activation might be associated with fibrosis regulation [10]. Therefore, we quantified the total collagen of the hearts used for endothelin receptor determination. We did not find any correlation between the total collagen content and the ET_A_ or ET_B_ receptor density, respectively. Thus, our data do not support a correlation of endothelin receptor regulation and cardiac fibrosis in human heart failure. However, fibrosis regulation might be more complex and a comparison between endothelin receptor regulation and myocardial collagen content is not sufficient to rule out an impact of endothelins for fibrosis development in human heart failure [34]. However, the role of endothelin receptor activation for myocardial fibrosis development was beyond the goals of this study.

In summary, conflicting data of endothelin receptor expression in DCM hearts exist which might be due to the control hearts used in a given study. In our patient population, we could not detect any influence of VAD-unloading on ET_A_ or ET_B_ receptor density, respectively.

## Supporting Information

S1 Fig Representative competition of ET-1 on 10,000 cpm [^125^I]-ET-1. All experiments were performed in duplicates. The dashed lines represent the total binding (TB) and the non-specific binding (NSB).
(TIF)

S2 Fig Representative biphasic competition of BQ-123 on 10 000 cpm [^125^I]-ET-1. All experiments were performed in duplicates. The dashed lines indicate the portion of each receptor subtype or represent the total binding (TB) and the non-specific binding (NSB), respectively.
(TIF)

S3 Fig Correlation of receptor expression with the collagen quantity. Myocardial tissue was analyzed for ET_A_ **(A)** and ET_B_ **(B)** receptor density and hydroxyproline content. The spearman correlation coefficient is indicated. The graph contains NF, pre-VAD and post-VAD samples.
(TIF)

S4 Fig Influence of VAD implantation on Plasma Big-ET1 level. Plasma Big-ET1 was measured using a commercially available ELISA according to manufacturer’s conditions (Biomedica). Plasma Big-ET1 in patients supported by LV assist devices. Plasma Big-ET1 is significantly lower after 28 ± 11 days (mean ± SD) VAD-support (p < 0.05).
(TIF)

S5 Fig **Correlation of endothelin receptor density and Plasma Big-ET1 level at the time of (A) VAD implantation and (B) after VAD support.** There is no evidence for a correlation of myocardial ET_A_ or ET_B_ receptor density and plasma Big-ET1 at VAD implantation or after VAD support, respectively. Of note, post VAD plasma samples were measured 28 ± 11 days (mean ± SD) after VAD implantation whereas the receptor density was measured at the time of heart transplantation.
(TIF)

S6 Fig Literature evaluation of ETR binding data. Ratios of the means for relative endothelin receptor expression in human DCM hearts plotted against the endothelin receptor density in NF control hearts derived from the literature (see [22, 24–29] in the main manuscript). (**A**) The published ET_A_ receptor density regulation appears to be dependent on the receptor density measured in rejected donor hearts (nonlinear regression; one phase decay; r^2^ = 0.8). Significant regulation of ET_A_ was only detected in studies using control hearts with receptor densities below 42 fmoles/mg. (**B**) In contrast the regulation of ET_B_ receptor density is not dependent on the receptor densities in control myocardium. (●) ratios of the means reflecting significant changes compared to controls; (○) ratios of the means reflecting no significant changes compared to controls. Of note, results of this study were given as large symbols.
(TIF)
